# Chitosan: Properties and Its Application in Agriculture in Context of Molecular Weight

**DOI:** 10.3390/polym15132867

**Published:** 2023-06-28

**Authors:** Ramón Román-Doval, Sandra P. Torres-Arellanes, Aldo Y. Tenorio-Barajas, Alejandro Gómez-Sánchez, Anai A. Valencia-Lazcano

**Affiliations:** 1 Tecnológico Nacional de México, Instituto Tecnológico del Valle de Etla, Oaxaca 68230, Mexico; sandra.torres071299@gmail.com (S.P.T.-A.); alejandrogomez@itvalletla.edu.mx (A.G.-S.); 2 Faculty of Physical Mathematical Sciences, Meritorious Autonomous University of Puebla, Puebla 72570, Mexico; aldoyair.tenoriobarajas@viep.com.mx; 3 Tecnológico de Monterrey, School of Engineering and Sciences, Mexico City 14380, Mexico

**Keywords:** Chitosan, crustaceans, sustainable agriculture, antimicrobial

## Abstract

Chitosan is a naturally occurring compound that can be obtained from deacetylated chitin, which is obtained from various sources such as fungi, crustaceans, and insects. Commercially, chitosan is produced from crustaceans. Based on the range of its molecular weight, chitosan can be classified into three different types, namely, high molecular weight chitosan (HMWC, >700 kDa), medium molecular weight chitosan (MMWC, 150–700 kDa), and low molecular weight chitosan (LMWC, less than 150 kDa). Chitosan shows several properties that can be applied in horticultural crops, such as plant root growth enhancer, antimicrobial, antifungal, and antiviral activities. Nevertheless, these properties depend on its molecular weight (MW) and acetylation degree (DD). Therefore, this article seeks to extensively review the properties of chitosan applied in the agricultural sector, classifying them in relation to chitosan’s MW, and its use as a material for sustainable agriculture.

## 1. Introduction

Chitosan is a mucopolysaccharide and, in its natural form, a linear polymer of 1,4-glycosidically linked glucosamine (2-amino-2-deoxy-D-glucopyranose). However, in practice, it also typically contains residues of acetylglucosamine (2-acetamido-2-deoxy-D-glycopyranose). A substance is categorized as chitosan if the concentration of these acetylglucosamines is lower than 50% and chitin if the concentration is 50% or more. After cellulose, chitin (β-1,4-N-acetyl-D-glucosamine) is the most abundant natural biopolymer of at least 1010 tons existing in the biosphere. Also, chitin is a component of the exoskeleton of crustaceans, insects, and the cell walls of fungi [1,2]. The importance of chitin as a source of carbon and nitrogen for marine species and its effects on the ecosystem within the ocean has recently come to light. The primary resources used for the industrial production of chitosan and chitin are marine crustaceans, shrimp, crab, and squid bone plates [3]. Chitin and chitosan are nontoxic, biocompatible, and biodegradable polymers. They have enormous unexplored potential to make sustainable agriculture a reality.

Chitosan is one of the most studied natural polymers in different application areas. For example, in tissue engineering, it is used due to its biocompatibility, antimicrobial, hemostatic, and biodegradability properties. Likewise, it can be used as a base material for making electrospun fibers, sponges, hydrogels, and membranes [4,5,6]. The use of chitosan as a base material with other materials such as other biopolymers, micro and nanoparticles, and active ingredients increases their biocompatibility properties as well as their mechanics, promoting a favorable microenvironment for cell regeneration [4,5,6,7,8]. Chitosan is also used as food packaging due to its nontoxicity, antimicrobial, bactericidal, permeability, and mechanical properties [9,10]. Chitosan is used in bioremediation because it is a natural active absorbent that can be used to absorb heavy metal ions [11,12]. Because chitosan has active groups, it can be modified with other materials such as humic acids, carbon nanotubes, and the algae Ulva lactuca, which increases its efficiency in absorbing metal ions [13,14]. In fact, porous carbon microspheres obtained from chitosan are used to manufacture new-generation supercapacitor electrodes [15]. Furthermore, one area where chitosan has much potential due to its multiple properties is agriculture.

Chitosan induces callose formation, acts as a proteinase inhibitor and helps in phytoalexin biosynthesis. Chitosan applied topically enhances stomatal conductance, increases abscisic acid (ABA) content, and reduces perspiration in plants without altering their height, leaf area, root height, or biomass. It can also be used to coat seeds for fruits, vegetables, nuts, and cereals. It increases proline and sugar contents, thereby changing the seed plasma membrane permeability. It also improves the activity of the enzymes peroxidase, phenylalanine ammonia-lyase, tyrosine ammonia-lyase, and catalase. Chitosan acts as an antifungal, antiviral, and bionematicidal agent. Chitosan acts as a carrier promoting slow-release of fertilizers. It also improves the water retention capacity of the soil. Chitosan also has the best chelating properties, is able to remove heavy metals and dyes, controls algal contamination from lakes, and acts as a soil conditioner [16].

This review presents a plethora of studies on the use of chitosan in the agricultural sector. We classify chitosan depending on its molecular weight and present its own chemical, physical, and biological characteristics. We further discuss its antifungal, antiviral, and antibacterial properties as well as how to use it to alleviate abiotic stress, as a growth promoter, and to enhance the physiological characteristics of plants. These properties were discussed as they relate to the molecular weight of chitosan. This review presents sources, characteristics, and properties of chitosan and their relationship to its application in agriculture. The objective of this review was to gather the knowledge generated so far and explore how the molecular weights of chitosan and its ability to combine with other materials influence its application in the agricultural sector. So far we have not found a proposal like the one presented in this work, so it will be of great interest to the reader who works in the agriculture area.

## 2. Chitosan: Structure and Natural Origins

**Figure 1:**
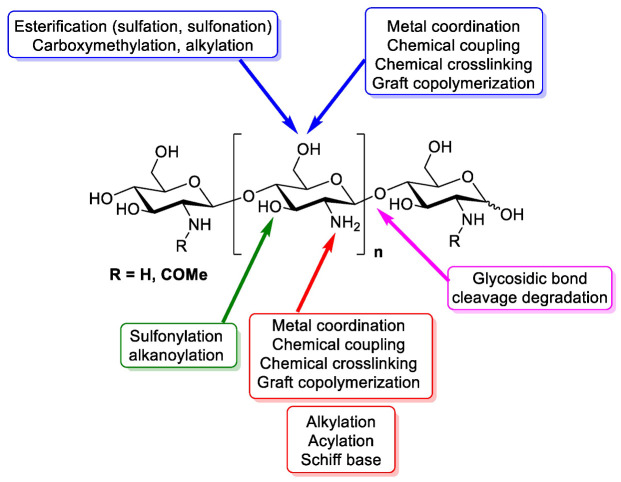
Design of chitin and chitosan structure, chemistry and functional groups that are able to be modified [19].

Chitosan is a linear polymer composed of two subunits, D-glucosamine and N-acetyl-D-glucosamine, that are joined by 1,4-glycosidic bonds. The three rings of the chitosan molecule make up its overall structure (Figure 1). It has three functional groups including amino groups, primary and secondary hydroxyl groups, which make its chemical modification easy. Additionally, these functional groups influence chitosan’s solubility and mechanical properties. Furthermore, chitosan possesses -1,4 glycosidic linkages. In acidic aqueous environments, chitosan is more soluble than chitin. The primary reason for chitosan’s solubility is the protonation of –NH${}_{2}$ at the C–1 position of the D-glucosamine repeat unit, which transforms the polysaccharide into a polyelectrolyte under acidic conditions. Due to its solubility properties, nearly all areas of human life and health (agriculture, medicine, process engineering, and industries) can benefit from using chitosan. Chitosan can be directly extracted from a variety of fungus; it can also be produced by extracting chitin and then passing it through a deacetylation process. In the fungal kingdom, chitin is a polymer that is made more frequently than chitosan, being produced by zygomycetes, ascomycetes, basidomycetes, deuteromycetes and phycomycetes. In contrast, chitosan is exclusively found in the cell walls of a few taxa of fungi, particularly, zygomycetes. Extraction of chitosan from fungal biomass is greatly advantageous as it can be done at any time and is not prone to seasonal changes. A process similar to that used to extract chitosan from crustaceans is used to extract chitin from fungi whose cell walls are the sole source of it. Therefore, using only fungi that currently produce the desired product is more cost effective [17]. Another very important and abundant source of chitin comes from the exoskeletons of crustaceans; mainly from a variety of marine crustaceans such as shrimp, crabs, and lobsters. Currently, the main source of chitin comes from waste from the shrimp industry, where exoskeletons are obtained to obtain chitin and calcium. Chitosan is obtained through a chemical process of N-deacetylation, where the cationic nature of chitosan is owed to the free amino group left by the partial removal of the acetyl group of chitin [18]. The diverse sources of chitosan and chitin are shown in Table 1.

## 3. General Characteristics of Chitosan

The source and method of extraction have a direct impact on the properties of chitosan. The primary properties that determine the efficacy and scope of applications for chitosan include particle size, molecular weight, crystalline structure, level of deacetylation, and surface area.

The molecular weight (MW) of chitosan depends on how many monomeric units are in the biopolymer. Viscosity and solubility are two properties that are impacted by MW, thus their control, assessment, and modification are crucial. The MW of chitosan typically varies from 20 to 1200 kDa [41]. It can be determined using light scattering and high-performance liquid chromatography, but the most popular and straightforward method is the viscosimetric method.

The deacetylation degree (DD) is another important factor that influences the characteristics and applications of chitosan. The relation between units of 2-acetamido-2-deoxy-D-glucopyranose is known as DD. When the biopolymer only contains monomeric forms of 2-amino-2-deoxy-D-glucopyranose, the degree of deacetylation is 100 percent, and the biopolymer is totally deacetylated. When the percentage of 2-amino-2-deoxy-D-glucopyranose units reaches 50%, the polymer is typically known as chitosan and turns soluble in aqueous acidic environment. The properties and uses of chitosan are influenced by the deacetylation degree, much like MW [42].

Crystallinity, a property of chitosan, reflects the proportion of the biopolymer’s crystalline and its amorphous fractions. This property is measured by the crystallinity index (CI). Chitosan is a polymorphic biopolymer and is semi-crystalline in its solid form. It has an orthorhombic unit cell with two antiparallel chains without water molecules. The source and preparation methods influence the crystallinity of chitosan. The highest value of crystallinity is shared by chitin (0% deacetylated) and 100% fully deacetylated chitosan. Quantifying CI is essential since it influences how chitosan swells as well as its porosity, water absorption and moisture retention. The relation between X-ray diffraction characteristic peaks can be used to determine CI [43].

The surface area and size of particles are two of chitosan’s most crucial characteristics. The porosity of chitosan is related to the pore size distribution and volume of its pores, which in turn depend on the source and extraction process. Due to its nonporous nature, chitosan flake or powder has a surface area less than 10 m${}^{2}$/g. In most applications, particles lower than 1 mm is often used. Chitosan applications like adsorption and enzyme immobilization require several accessible sites and a porous structure; therefore, surface area and particle size are crucial factors. The nonporous nature of chitosan necessitates several modifications to increase its surface area. In fact, precise measurement of the size of the particles and its surface area is crucial. Nitrogen adsorption–desorption isotherms using the BET method are typically used to determine surface area. Particle size can be determined using a particle analyzer, sieving tests, or scanning electron microscopy [44].

Due to the diversity associated with its chemical structure, chitosan is a very attractive compound. Two methods to express this diversity are the molecular weight, which ranges from oligo-chitosan to high, medium, or low molecular weight, and DD, which ranges from acetylated chitosan to partially deacetylated chitin [45]. Three basic types of chitosan may be categorized according to their molecular weight ranges: low molecular, weight chitosan (LMWC, less than 150 kDa), medium molecular weight chitosan (MMWC, 150–700 kDa), and high molecular weight chitosan (HMWC, >700 kDa) [46]. The preparation process and raw material sources influence chitosan’s molecular weight. Native chitin typically has a molecular weight of over 1,000,000 Da, whereas industrial chitosan products typically range from 100,000 to 1,200,000 Da. Generally, shear stress, dissolved oxygen, and high temperatures may all cause chitosan to deteriorate. For instance, chitosan undergoes heat degradation at temperatures higher than 280 °C, which causes polymer chains to break down quickly and reduce the molecular weight. Additionally, when EDTA is used, maximum depolymerization brought on by the use of strong or high-temperature acids such sulfuric acid, acetic acid, and hydrochloric acid leads to molecular weight alterations with little degradation. Chromatography, light scattering, and viscosimetry are some of the techniques that can measure chitosan’s molecular weight [47].

## 4. Properties

### 4.1. Low-Molecular-Weight Chitosan

Generally, the LMWC product following the degradation process by any method is a complex mixture of various DD and MW and frequently exhibits a significant MW distribution (MWD). Usually, these characteristics have a considerable impact on the properties of LMWC, especially its biological activities. Furthermore, it is challenging to pinpoint precisely the type of chitosan molecules responsible for the observed properties when using heterogeneous chitosan mixtures. Thus, these three characteristics (MWD, MW, and DD) must be established before any other characterizations of LMWC are performed.

#### 4.1.1. Physicochemical Properties

The physical and chemical properties of LMWC are mostly the same as those of the original chitosan. They include the following: it is a linear amino polysaccharide with high nitrogen content, it is a weak base with deprotonated amino groups as nucleophiles (pKa = 6.3), it is able to form hydrogen bonds between molecules; it has highly reactive groups for crosslinking and chemical activation; it forms salts with organic and inorganic acids, it has chelating and complex properties, and it has ionic conductivity as polyelectrolytes (pH < 7). Furthermore, the low MW yields high solubility, aqueous solutions with little viscosity, and increased permeability, giving it a huge potential for use in the food, medical, and agricultural industries. Partial hydrolysis of chitosan shows that LMWC has less crystalline structure than HMWC. The thermal, mechanical, and permeability characteristics of chitosan membranes are affected by the low MW. LMWC has higher permeability, lower melting point and higher tensile strength than HMWC. Similarly, the thermostability of chitosan decreases as its MW is reduced.

#### 4.1.2. Biological Properties

The biological properties of LMWC are similar to those of chitosan derivatives with amine, hydroxyl and acetylated amine groups. This leads to a variety of biological properties such as biocompatibility, nontoxicity, biodegradability, antimicrobial activity (against fungi, bacteria, and viruses), mucoadhesive, antioxidant activity, antitumor, hypolipidemic, blood anticoagulant activity, and hypocholesterolemic activities [48,49,50,51,52]. These bioactivities heavily rely on the LMWC’s molecular weight, MW distribution, and DD. Among its properties, chitosan’s chain length and dispersion are thought to be the main determinants of its biological activity. In contrast to the long chitosan strands, the short low molecular weight chitosan chains with a restricted distribution are practically easier to absorb on the surface of substrates [50,51]. LMWC is more biologically active than chitooligomers and HMWC. However, most of its bioactivity processes are still debatable. This might be because the LMWC mixture used included different molecular weight and MW distribution values that were primarily derived from the deteriorating mechanism [52,53]. To fully understand the bioactivity mode of action, a highly pure LMWC of a specified size is required. Numerous chromatographic techniques can be used to separate the heterogeneous LMWC molecules [52].

The degree of deacetylation of LMWC influences biological activities. An increased DD typically causes an increased antibacterial activity. For instance, LMWC with a higher DD (92%) had more antibacterial activity against *Staphylococcus aureus* and *Escherichia coli* LMWC with a lower DD (80%) [54]. The effectiveness of antioxidant activity of LMWC against 1,1-diphenyl-2-picrylhydrazyl, hydroxyl, superoxide, and carbon-centered radicals, depends on its degree of deacetylation. It can be described based on the chitosan scavenging mechanism. To form a stable molecule, a free radical must interact with the H^+^ ion from the (NH${}_{3}^{+}$) ions produced as a result of NH${}_{2}$ groups receiving H${}^{+}$ ions by the solution [41,55].

There are several previous studies on chitosan’s cytotoxicity, particularly regarding the connection between cytotoxicity and molecular weight. However, the findings of these studies remain controversial. Some studies suggest that chitosan is toxic with toxicity depending on the MW and DD. Other studies, however, suggest that chitosan’s toxicity is minima [46,56].

### 4.2. Medium-Molecular-Weight Chitosan

Medium molecular weight chitosan is soluble in weak acid solutions. This limits their usage compared to the antimicrobial capacity of acid- and water-soluble chitosan with different degrees of deacetylation and viscosities.

#### 4.2.1. Physicochemical Properties

As chitosan is widely used in films, there are studies focused on the effect of chitosan MW on film properties. For example, Bof et al. [57], investigated chitosan’s molecular weight in relation to starch-composite film characteristics. They established that the MMWC-based filmogenic solutions have a pseudoplastic behavior, boosting the film’s apparent viscosity and consistency index. Additionally, Doval et al. [58] reported the production of composite films made of chitosan, graphene oxide (GO), and pullulan. Their findings revealed that adding GO increases the roughness of the film while maintaining high graphene oxide dispersion and that both the strong interactions between chitosan and pullulan (300 kDa, 82% DD) as well as the production of hydrogen bonds with graphene oxide and chitosan appear to be the principal factors that lead to the production of the composite film. As its DD, elastic modulus, and tensile strength peak at 1 μg mL${}^{-1}$ GO content, it appears that pullulan-chitosan blended composite films CF-Pul/Chi 50/50 + 1 µg mL${}^{-1}$ GO have suitable properties for secure films and for uses in food, cosmetics, and pharmaceutics. Additionally, Strupiechonski et al. [59] produced thin films of CS/AuNP by chemically reducing HAuCl${}_{4}$ in sodium citrate and chitosan-based films (CS) solutions. They discovered that depending on the HAuCl${}_{4}$ concentration and water content, nanocomposites of various conductivities could be produced, which could change the characteristics and sensitivity of sensors.

#### 4.2.2. Biological Properties

The antioxidant performance of chitosan is influenced by several factors, including content, DD, antioxidant enzymes, and MW. MMWC exhibits stronger antioxidant activity than LMWC and HMWC. For example, Tokatli et al. [60] investigated the effects of chitosan at varying levels of MW and deacetylation on cherry preservation focusing on antioxidative properties, total phenolic composition, cherry color, ascorbic acid content, total anthocyanin composition, toughness, and total pectin composition. Both ascorbic acid content in cherries and the antioxidant capacity of chitosan varied with the level of MW and deacetylation, with the highest antioxidant capacity occurring in chitosan with 81.22% deacetylation and of 273 kDa MW at 4 °C.

The potential of MMWC films has been confirmed by several investigations. Dotto and colleagues [61] investigated the application of chitosan filmogenic solutions to prolong the microbiological storage life of the papaya fruit kept at room temperature. They discovered that chitosan solutions with a medium molecular weight (150 kDa) are better able to preserve papaya fruits and increase their shelf life by four to seven days.

### 4.3. High-Molecular-Weight Chitosan

HMWC is frequently used in quaternized chitosan films mainly due to its solubility in water and capacity to scavenge free radicals. Because of its influence on polymer aggregation and phase separation, HMWC is additionally used in high-performance cells [62].

#### 4.3.1. Physicochemical Properties

Generally, the DD and MW of chitosan influence its solubility. The higher the MW of chitosan, the more the inter- and intra-molecular hydrogen bonds inside the polymer chain, causing entanglement of its molecular chains and difficulty in dissolving [63]. Consequently, HMWC exhibits lower solubility in water than LMWC [64]. Additionally, HMWC is rarely soluble in aqueous solutions at neutral pH as chitosan has a weak basic property with a pKa value of 6.5 [48]. Similarly, chitosan molecules with high molecular weight find it difficult to pass through cell membranes due to their high viscosity [63].

Additionally, several previous studies have focused on how chitosan MW affects film properties. For example, Bof et al. [57] demonstrated that filmogenic solutions based on HMWC exhibited a Newtonian pseudoplastic fluid, enhancing the film’s apparent viscosity as well as consistency index. This study also looked at the effects of chitosan MW on starch-composite film qualities and found that 1600 kDa (82% DD) chitosan polymer has outstanding rheological characteristics and great mechanical resistance. They concluded that HMWC can be used as an antibacterial coating on various food products [65,66].

#### 4.3.2. Biological Properties

HMWC is highly viscous and is used in extended dosage form in oral tablets to prolong pharmacological action, improve treatment effectiveness, and reduce adverse effects [67]. García et al. [68] investigated the effects of chitosan MW on *Candida parapsilosis*, *C. tropicalis*, and *C. albicans* and concluded that HMWC exhibits high adsorption on cell walls, resulting in the covering of cell walls, weakening, rupture, and leaking of cell membranes. Additionally, 746 kDa chitosan against *Pseudomonas fluorescens* and *E. coli* found to be more efficient for Gram-negative bacteria than 746 kDa chitosan versus *Vibrio parahemolyticus* as well as *Salmonella typhimurium* [69].

According to Aranaz et al. [19], larger chains of HMWC create more intramolecular hydrogen bonds than those of LMWC, which makes the reactive groups less accessible. Consequently, HMWC samples exhibit a less noteworthy antioxidant activity than those of LMWC. Furthermore, Kou et al. [64] stated that high-molecular-weight chitosan typically exhibits less significant bioactivities than low-molecular-weight chitosan. Table 2 shows a resume of the properties described in this section.

## 5. Applications in the Agricultural Sector

Chitosan is a versatile material with antibacterial properties that are effective against fungi, bacteria, and viruses. Chitosan also stimulates plant defense mechanisms. Because of its broad-spectrum antibacterial properties, chitosan has been used to slow down or stop the spread of diseases and to boost plant defense mechanisms. Chitosan is an antimicrobial compound with great potential to control pathogenic plant diseases due to the interaction of its antimicrobial and eliciting characteristics [70]. Chitosan is also involved in the stimulation of secondary metabolite formation, which strengthens the immunological defense mechanisms of plants [71].

### 5.1. Antimicrobial Properties

The type of microbe, DD, and MW, in addition to inoculant concentration, temperature, culture medium, and pH, are all factors that affect chitosan’s antimicrobial efficacy [63]. Chitosan may inhibit various plant diseases [70]. The types of microorganisms that are susceptible to chitosan are divided into sensitive and resistant fungi and both Gram-positive and Gram-negative bacteria [72].Chitosan inhibits several harmful bacteria, including *Xanthomonas*, *Pseudomonas syringae* [73], *Agrobacterium tumefaciens*, and *Erwinia carotovora* [74]. Nevertheless, chitosan appears more effective against fungi than against bacteria.

#### 5.1.1. Low-Molecular-Weight Chitosan

LMWC is a promising molecule for use for treating plant diseases due to its high solubility in water and simplicity of processing for aquaculture systems [75]. LMWC may be able to permeate the cell wall of bacteria, disrupting their regular physiological metabolism, or even directly affecting their genetics, flocculating, and degenerating intracellular components, inhibiting bacterial reproduction, and ultimately causing the death of microorganisms [70]. As Gram-negative bacteria contain very thin cell walls, LMWC can easily penetrate the cells and destroy their genetic material, thereby acting more effectively against these bacteria [76]. Additionally, chitosan with a molecular weight of less than 10 kDa has a stronger antibacterial effect than that with a large molecular weight because it is more soluble. Several studies have noted that chitosan polymers with lower molecular weights and higher levels of acetylation are more effective in slowing down and inhibiting the growth rate of microorganisms than those with higher molecular weights [77]. For example, it has been demonstrated that Rhizopus stolonifera is most inhibited by LWMC [78]. Additionally, *Pseudomonas syringae pv*. Tomato, a bacterium that causes tomato’s bacterial speck, shows no resistance against chitosan with a deacetylation level of 78% and an average MW of 70 kDa [73]. Moreover, according to Badawy et al. [79], LMWC appears to be effective in opposition to bacteria of *E. carotovora* and *A. tumefaciens*. Additionally, Ortega et al. [80] investigated the effect of chitosan’s MW on its effectiveness against bacteria of *P. aeruginosa* and *P. oleovorans* strains. According to the findings, HMWC shown a 64.57% inhibitory percentage and LMWC had a 72.52% inhibitory percentage. Additionally, *E. carotovora*, *A. niger* and *R. stolonifer*, three major plant pathogenic fungi and bacteria respectively, are considerably inhibited by the synergistic effect of citral and LMWC [81].

#### 5.1.2. Medium-Molecular-Weight Chitosan

MMWC exhibits high antibacterial activity and its mode of action is thought to be as follows: by coating the cell surface, MMWC prevents nutrients from getting to the microbial cellular membrane, consequently causing cell lysis [82]. Studies looking at the antibacterial activity of chitosan at various MW against *S. aureus* and *E. coli* revealed that the antimicrobial effect of chitosan of MW < 300 kDa against *S. aureus* was boosted as the MW raised, whereas the impact on *E. coli* was diminished [83]. In addition, Di Piero and Coqueiro [84] reported that inoculation. They discovered that MMWC inhibited and prevented the bacterium *X. gardneri* from staining tomatoes [19]. In another study on how MW affects antibacterial effectiveness, only MMWC exhibited antimicrobial activity against multiresistant isolates of Gram-positive *S. aureus*, *L. casei*, and Gram-negative *E. coli* [85].

#### 5.1.3. High-Molecular-Weight Chitosan

The formation of a thick polymer covering on bacterial cell surfaces is thought to be possible using HMWC. This is thought to be the mechanism by which HMWC exerts its antibacterial effects. Cells exposed to chitosan display changed exterior membranes, the outside becomes coated with many vesicles as well as an extra layer of material, suggesting that the cell envelope becomes thicker. The thickened cell envelope hinders the transport of excreted metabolites outside the cell and the uptake of nutrients. Therefore, the production of polymeric films that damage the physiological metabolism of bacteria is possibly the basis for HMWC antibacterial activity [70]. HMWC exhibits a better bactericidal effect by generating a covering that encloses bacterial cells, partly because Gram-positive bacteria have cell walls that are thicker than those of other bacteria [47]. To confirm this, Abdellatef et al. [78] showed that HMWC exhibits strong efficacy against *Alternaria solani* and *Fusarium oxysporum f.* sp. *vasinfectum*.

Additionally, Li et al. [86] proposed the amino protonation and subsequent cationic generation on the molecular side chain of ultrahigh MW chitosan molecules in acidic medium. They found that ultralong molecular chain of HMWC allowed it to bind and cover *Staphylococcus aureus* and *E. coli* cells before the cells became ruptured and progressively degraded.

### 5.2. Antiviral Properties

Despite not being a true part of the virus, chitosan inhibits viral infections in plants. Chitosan can prevent viral infection by inducing a hypersensitive response, which decreases the survival of phage bacterium cells, neutralizing the infectivity of phage particles, and limiting the multiplication of virulent phages [87,88]. Similar to its antibacterial and antifungal properties, chitosan’s antiviral activity is influenced by various variables, including its concentration, molecular weight, and degree of deacetylation [89]. The few published studies on chitosan’s antiviral action indicate that it can inhibit the multiplication of viruses and viroids, hence restricting their replication [75].

#### 5.2.1. Low-Molecular-Weight Chitosan

Chitosan’s antiviral effectiveness against viruses in microbes and plants has been studied, there’s evidence suggesting the potential of LWMC against viruses. Regarding this, Faqir et al. [90] shown that LMWC reduces the development of localized necrosis caused by the mosaic virus of tobacco by 50% to 90%. Additionally, oligo-chitosan LMWC is effective at controlling M. incognita and reducing tobacco mosaic virus (TMV) infection in tobacco [91]. In another study examining the antiviral effects of chitosan against alfalfa mosaic virus (AMV) in beans, the findings revealed that LMWC treatment of a bean leaf’s lower surface produced resistance to AMV on that leaf’s upper surface. Resistance was induced in upper leaves by treating the lower leaves, and resistance was induced in the treated half of a leaf as well as in the untreated half [92]. A previous study reported that a systemic pathogen of the bean mild mosaic virus became more resistant to chitosan as its MW decreased [79]. Additionally, Davydova and colleagues [93] depolymerized chitosan by enzyme and chemical-based hydrolysis to produce chitosan with various MW and DD. Additionally, chitosan derivatives (2.0–17.0 kDa) reduced local necrotic lesions in the *Nicotiana tabacum* plant by the systemic TMV by 50–90%.

The improved capacity of LMWC to penetrate the tissues of the leaf epidermis may, in essence, be responsible for a boost in the antiviral action.

#### 5.2.2. Medium-Molecular-Weight Chitosan

To date, there’s scant knowledge of the antiviral effects of MMWC. Foliar spraying of chitosan (600 kDa) stimulated the production of defense genes in chili plants and prevented the accumulation of pepper mild mottle virus and cucumber mosaic virus [94].

#### 5.2.3. High-Molecular-Weight Chitosan

According to several research, HMWC has exceptional antiviral action. For instance, according to El Sayed et al. [95], at the recommended concentration (1:1 dilution), LMWC and HMWC reduced the amount of gall by 90 and 93%, respectively. Additionally, Silva et al. [96] demonstrated that high-molecular-weight-chitosan (HMWC) is more efficient than LMWC in reducing Pine Wilt Disease, which is brought by *Bursaphelenchus xylophilus* (pine wood nematode). Moreover, localized necrotic lesions caused by TMV inoculation on tobacco plants were considerably suppressed by enzymatic breakdown of HMWC by *Aspergillus fumigatus* fungal chitinases [97].

### 5.3. Antifungal Properties

**Figure 2:**
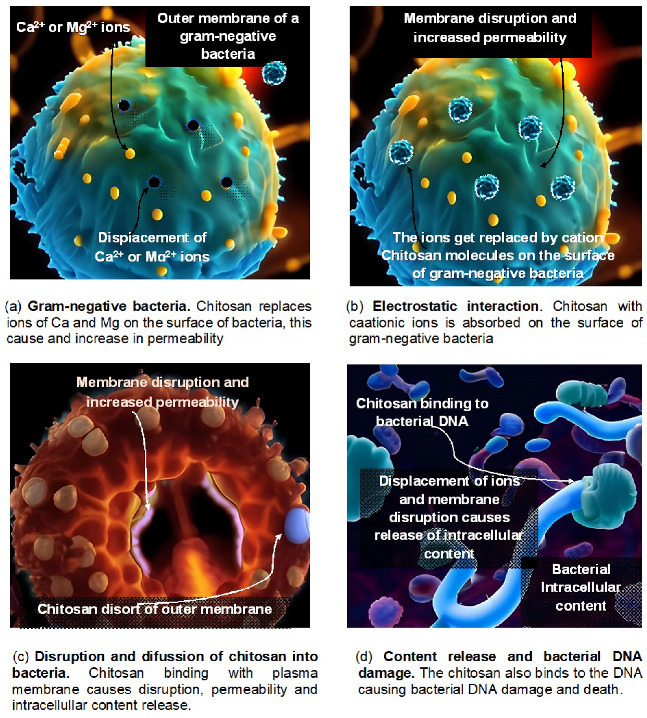
Effects of chitosan on bacteria.

As it prevents the growth of many harmful fungi in vitro, including *Colleotrichum gleosporoide*, *Alternaria alternata*, *Rhizopus stolonifera* and *Botrytis cinerea*, chitosan exhibits broad-spectrum antifungal effects. Inhibition was observed in the synthesis of components for fungal virulence at several phases of pathogen development, including sporulation, mycelial growth, germination, and spore viability [98]. Antifungal action occurs in vivo in numerous plant pathogen systems, for example, in the pear against *Physalophora piricola* and *Alternaria kikuchiana* [99], in the strawberry and grapevine agains *Botrytis cinerea* [100,101], and in the dragon fruit against *Colletotrichum gleosporoides* [102]. Using observations from pathogenicity testing and TEM, it was possible to further demonstrate the antifungal effect of chitosan in rice against *Rhizoctonia solani* [103]. Figure 2 shows a sequence of one of the action mechanisms of chitosan in bacteria.

#### 5.3.1. Low-Molecular-Weight Chitosan

A recent study examined the antifungal mechanism of LMWC using about 4600 nonessential gene deletion mutants of *Saccharomyces cerevisiae*. It was discovered that 31% of the 107 chitosan-sensitive mutants had deletions in genes predominantly responsible for protein synthesis [104]. Ippólito et al. [105] showed that LMWC added to an ineffective dosage of Mancozeb resulted in a highly effective treatment for late blight. LMWC and commercially available synthetic fungicides have a synergistic impact against *B. cinerea*, *A. brasicicola*, and *Muocor piriformis* [106]. The citrus green mold induced by *Penicillium digitatum* was successfully controlled by chitosan derived from shrimp shells [107], but the mechanism of action is still unclear. Additionally, in vitro tests using the fungus *B. cinerea* sshowed that chitosan’s antifungal effectiveness increased as its MW decreased. Similar findings were made with *Aspergillus niger*, where LMWC had the highest antifungal efficacy. *B. cinerea* and *P. expansum* revealed a notable inhibition of their mycelial growth when exposed to LMWC [108]. Another study found that *A. ochraceus*’s spore germination and mycelium growth were inhibited by chitosan with a DD of 93% and an average MW of 100 kDa [109]. The study further showed that LMWC can also cause striking modifications to the inner microstructure and exterior morphology of *A. ochraceus*. Moreover, LMWC impeded the growth of *P. expansum*, *B. cinerea*, and *Rhizopus stolonifera* [110]. Furthermore, Nguyen et al. [111] demonstrated that the use of LWMC solutions on rice plants had significant impacts on the biological activity of living tissues, common brown-backed rice hoppers, and fungicidal properties. Also, treating tomato plants with LMWC (70 kDa) improved their resistance to nematodes such as *Meloidogyne* spp. LMWC is effective against *Fusarium oxysporum*, *B. cinerea*, and *P. debaryanum* [79] as well as spores of *P. infestans* and *F. eumartii* spores [75].

In many plants, chitosan and its variants have a great variety of eliciting compounds. Chitosan treatment induces chitinase and glucanase enzymes in some crops. For instance, LMWC (5000 Da) could activate the production of ROS species in rice seedlings and promote phytoalexin as well as other pathogenesis-related chemicals, chitinase, -glucanase, and lipoxygenase [112]. Chitosan is also used as a protective covering on maize seeds in abiotic stresses, enabling seedlings to establish defense responses against the fungi *F. moniliforme* and *A. flavus*. The effectiveness of chitosan is reflected mostly in the reduction or lack of diseases in the various seedling components [113]. Studies [114,115] on the synergistic antimicrobial activities of chitosan mixtures and chitosan–copper combinations confirmed that the chitosan treatment reduces myco-toxin generation in plants and has direct antifungal effects on the vegetative growth of *F. graminearum*. The antimicrobial efficacy of chitosan-CuO nanocomposites made with olive leaves extract are environmentally friendly, cost-effective, biogenic molecules with antifungal activity. Furthermore, Mahdavi Rahimi. Furthermore, Mahdavi & Rahimi [116] discovered that *Carum copticum* plants treated with LMWC lengthened the shoots and roots and increased their dry weight and relative amount of water during salinity stress. In a different study, LWMC (50 kDa) increased protein content and lowered antioxidant enzyme activities in ajwain seedlings and calluses subjected to salt conditions [117]. Additionally, Golkar et al. [118] showed that the total content of phenolics, flavonols, and flavonoids in the safflower callus as well as its antioxidant activity increased significantly as a result of chitosan and salicylic acid elicitation to mitigate the negative effects of salinity stress. Chitosan can make complexes containing nonnutrient elemental ions, such as a variety of heavy metals, because it contains functional amino and hydroxyl groups. Chitosan of various molecular weights, including those with 10,000 Da, 5000 Da, 690, and 1000 Da, reportedly reduces the harmful consequences of cadmium in consumable rape *Brassica rapa* L. grown hydroponically [119]. Chitosan treatment reduces myco-toxin generation in plants and has direct antifungal effects on the vegetative growth of *F. graminearum* [114]. Both molecular weight and molecular weight dispersity of chitosan are important for its antifungal activity [120].

#### 5.3.2. Medium-Molecular-Weight Chitosan

According to several studies, MMWC has a specific inhibitory impact on a few pathogens. On potato glucose agar, 15 different types of plant pathogenic fungus, including *R. cerealis*, *F. oxysporum*, and *Fusarium graminearum*, were examined by Faqir et al. (Table 3) [90] to assess their reactions to acid soluble chitosan and 2 types of water-soluble chitosan. The findings demonstrated that three different types of chitosan might be somewhat suppress fifteen different types of plant pathogenic fungus, unexpectedly demonstrating a better bacteriostatic effect on MMWC treatment.

However, the degree of inhibition depended on the physical and chemical properties of chitosan, the fungal pathogen concerned, and the resilience of the host’s immune responses [126,127].

Chitosan MW also affects how fungi respond physiologically and biochemically. For example, three isolates of *R. stolonifer* released more protein when chitosan MW increased [128]. Xing et al. [129] studied the antifungal and inducing characteristics of chitosan in inhibiting the growth of *Ceratocystis fimbriata* in sweet potatoes. The findings showed that MMWC (300 kDa) could reduce *C. fimbriata* spore germination and hyphae growth in vitro. Additionally, chitosan of 480–570 kDa average MW showed significant antifungal efficacy against *F. oxysporum* [130]. Wang et al. [131] also showed that chitosan of average MW 350 kDa and 90% DD directly reduced the development of *Sclerotinia sclerotiorum*’s and perhaps elicited a carrot defensive reaction. Another study demonstrated that MMWC (150 kDa) was able to protect potato plants from *Fusarium* spp. [132]. In fact, the wilt intensity caused by *Fusarium* spp. was considerably less severe in potato plants that received chitosan treatment (4.0 g/L of ethanoic acid distilled-water-solution) compared to pathogen-inoculated and untreated controls [132].

#### 5.3.3. High Molecular Weight Chitosan

The molecular weight of chitosan influences its antifungal activity for some phytopathogens but not for others. For instance, research on the phytopathogen *B. cinerea* Pers showed that chitosan with a large MW was more effective at inhibiting the growth of this pathogen than those that had a small MW [121]. Wang et al. [133] showed that using chitosan, azoxystrobin, and isopyrazam together is a potential agricultural strategy toward preventing Lasiodiplodie, which causes leaf spot disease in the kiwifruit. Furthermore, HMWC produces the greatest relative frequency of globose spores in *R. stolonifer* spores (2 mg/mL) and the highest variability in their form (elliptical-form factor) [87]. Additionally, HMWC demonstrated superior efficacy against *Valsa mali*, *Alternaria solani*, and *F. oxysporum f.* sp. *vasinfectum* [88]. Furthermore Wang et al. [134] showed that applying HMWC or LMWC to citrus (Murcott tangor) fruits greatly reduced postharvest deterioration caused by the fungi *Penicillium digitatum* and *Penicillium italicum*. In the Table 3 shows the inhibitory effects of chitosan on different fungal pathogens.

### 5.4. Chitosan’s Induction of Plant Protection Mechanisms

Because of its antibacterial, antifungal, and antiviral properties, chitosan is now regarded as a promising antimicrobial agent. It is now used in numerous agricultural activities. Since the 1980s, research on chitosan has shifted from its use as a general sewage treatment agent to its use as a seed coating agent, antistaling agent for fruits and vegetables, soil conditioner, and plant growth regulator, particularly in the fight against disease in agricultural production. Chitosan is an efficient inducer of plant systemic acquired resistance to infections in addition to being an antimicrobial agent. Chitosan has high effectiveness in disease control when used on plants in conjunction with biological control agents [70].

#### 5.4.1. Low Molecular Weight Chitosan

Utilization of chitosan in numerous pre- and postharvest treatments shows that it can activate the enzyme phenylalanine ammonia-lyase, boost total polyphenol levels in table grapes, inhibit storage gray mold growth, and activate polyphenol oxidase. It can also raise the overall amount of polyphenols in strawberries and enhance action of defense -related enzymes in bananas. Chitosan is effective in decreasing powdery mildew and elevating the overall polyphenol content [135]. Particularly, LMWC is a potent biotic elicitor that can induce plant defensive responses and trigger several pathways to boost the resistance of crops to pathogens [75]. LMWC (5 kDa) induces phytoalexin accumulation in plant tissue, lowers the overall amount, and altered the structure of free sterols [83]. Pests are negatively impacted by LMWC, in which it activates chitinase, glucanase, and lipoxygenases and increases the formation of reactive oxygen species. Additionally Fan et al. [136] reported that chitosan exhibited elicitor action by activiating the systemic and local defenses of tomato plants against the root-knot nematode *M. incognita*.

Furthermore, according to Ippólito et al. LMWC potentiates the effects of Mancozeb on potato crops [105]. Thus, the use of both substances may enhance chitosan’s ability to elicit plants and trigger natural defensive pathways. Comparable development tendencies in tomato seedlings were also reported. The host defense responses triggered by chitosan may also include glucanase activation, cell wall lignifications, phytoalexin biosynthesis, production of reacting oxygen species, and the potentiated accumulation of three chitinase isoforms found on potato leaflets. Moreover, rice seedlings treated with low molecula -weight chitosan produced a defense response against the fungus *Magnaporthe grisea*, which causes Rice Blast [110]. Similarly, Nguyen et al. demonstrated that LMWC can activate protective genes in rice plants via the octadecanoid pathway [111].

The application of chitosan of an average 5 kDa MW in *Betula platyphylla Suk* cell suspension cultures boosted the triterpenoids biosynthesis, indicating that chitosan increases chitinase activity in *Betula platyphylla Suk* cell suspension cultures, confirming the ability of chitosan to elicit defense mechanisms [137]. Foliar applications of chitosan in hydroponic pot trials over a range of MW (80% DD, 10,000 Da, 5000 Da, and 1000 Da, applied daily for one week) were able to reduce the cytotoxic activity of cadmium on the development and chlorophyll levels in edible rape leaves (*Brassica rapa* L.) [138]. Furthermore, treating seeds with LMWC (5–20 kDa) and spraying leaves with it every day induces resistance to *Phytophtora infestans* and *A. solani* [139]. Treatment of *C. annuum* with LWMC dramatically boosted endogenous H${}_{2}$O${}_{2}$, gene expression, and enzyme activity relevant to plant defenses, such as catalase 1 and phenylalanine ammonia-lyase [140].

#### 5.4.2. Medium Molecular Weight Chitosan

MMWC can also work as a trigger for plants to develop acquired systemic resistance to pathogens; nevertheless, studies in this area are limited. For example, chitosan of MW range of 50 kDa to 190 kDa can improve gas exchanges in diseased plants and simultaneously lower cucumber mosaic virus titers [141]. Similary, Liu et al. [142] showed that 1.25 g/L of chitosan of 350 kDa of average MW, effectively reduced *A. tenuissima*’s proliferation in vitro, acting as a natural elicitor triggering a host defensive response in potato tuber tissues. Additionally, chitosan of >85% DD and 350 kDa average MW induced reduction of the growth of the blue and gray mold and maintained kiwi fruit quality [143]. These chitosan benefits are most probably related to the stimulation of physiological and molecular defense -related responses in the kiwi fruit [143].

#### 5.4.3. High-Molecular-Weight Chitosan

Chitosan can be used widely to increase secondary metabolite yields and induce phytoalexin accumulation in plant tissue. Plants produce phytoalexins, which have antifungal and antioxidant properties, in response to pathogen challenges or when exposed to elicitors such as chitosan [70]. chitosan’s acetylation levels and MW affect phytoalexin synthesis. El Amerany et al. [144] showed that chitosan applied to shoots or roots of tomato plants led to a stronger plant response to wounding.

Additionally, Khalil and Badawy [145] and Radwan et al. [146], two separate research teams, examined at the advantages of chitosan on Meloidogyne incognita types in vitro and in a greenhouse environment. These results showed that HMWC provided a greater in vitro suppression of *M. incognita*’s second larval stage. As a result, in Consequently, treatment of soil contaminated with *M. incognita*, with HMWC under greenhouse conditions leads to a notable decrease in tomato root gall production and egg mass [145]. Moreover, soaking tubers with chitosan solution of different MW (between 20 and 970 kDa) improved their health [139].

### 5.5. Growth Promoter

**Figure 3:**
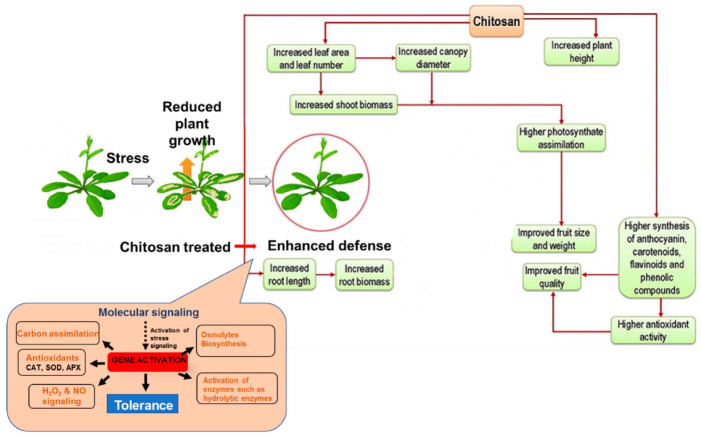
General description of chitosan-mediated plant growth regulation under stress conditions [91].

Composition, species, size, and the stage of growth of a plant, are factors that influence how chitosan functions [147]. Figure 3 shows a general description of chitosan-mediated plant growth regulation.

#### 5.5.1. Low-Molecular-Weight Chitosan

Literature suggests that the application of LWMC on plants affects positively in their growth. For example, rapeseed (*Brassica chinensis*) exhibits favorable germination index, seedling growth, and root length when coated with LMWC [83]. In a similar way, Darwis et al. [148] demonstrated that chitosan with an avg. MW of 141,000 Da and 14,000 Da respectively, both were effectively used on beans, potatoes, and chili to increase crop yields and reduce disease caused by virus, bacteria, and fungi. Furthermore, Nguyen et al. [111] discovered that the use of low-molecular-weight chitosan solutions improved the development and defense systems of rice plants by acting as an inducer. Additionally, chitosan as for MW of 124,000 Da and 66,400 Da, when treated at 31-, 45-, and 59-days following planting, a compound made by the authors with basic chitin deacetylation and acetylation degrees of 13.7 percent and 15.2 percent, correspondingly, increased the tuber size in 2 distinct potato cultivars (*Solanum tuberosum* L.) [149]. Furthermore, Mahdavi and Rahimi [116] reported that the germination percentage, germination rate, and seedling vigor index were all significantly impacted by the chitosan pretreatment of the seeds. In addition, low-molecular-weight chitosan known as “Fitosan” was created by deacetylating chitin inside a NaOH mixture at 90 °C over 8 h and subjecting it to a 75 kGy dose of gamma radiation. It was successfully tested in the field on a variety of plants, including rice, chili, potato, and soybean, to increase crop yields and control diseases [150].

#### 5.5.2. Medium-Molecular-Weight Chitosan

The mechanism of MMWC-induced plant growth remains mostly unknown. Chamnanmanoontham et al. [151] reported that MMWC (200–500 kDa) increased the dry and fresh weight of the leaves and roots, thereby boosting the vegetative growth in rice seedlings (at its seedling stage). Chitosan was first reported to have an eliciting effect on tomato (*Solanum lycopersicum* L.) and pea (*Pisum sativum* L.) plant growth and it has now been demonstrated that this action strengthens plant defenses against both biotic and abiotic stresses [152]. The potential of MMWC as an inducer of plant defense responses to abiotic stress has been demonstrated in several studies. For example, through promoting the build-up of phenolic chemicals as well as activating the antioxidant enzyme CAT, the administration of LMWC (50 kDa) and MMWC (190 kDa) considerably minimized the oxidative damage caused by salt in durum wheat seedlings [153]. Additionally, foliar application of MMWC soaked in various organic acids reduces the adverse effects of salt on tomato plants by enhancing photosynthetic pigments, raising osmoprotectant chemicals, nonenzymatic system ROS scavenging, antioxidant systems, and potassium contents [154].

#### 5.5.3. High Molecular Weight Chitosan

According to Chanratana et al. in greenhouse conditions, HMWC (at 75% DD) immobilized *M. oryzae* and accelerated germination and development of tomato seeds [155]. A different investigation by Salachna & Zawadziska [156] Soaking corms in chitosan solutions (2 kDa, 50 kDa, and 970 kDa) before planting resulted in plants that were taller, had more branches and leaves, blossomed earlier, had more flowers, and also produced more corms and HMWC appeared to be the best choice for enhancing these qualities [156]. Furthermore, Chookhongkha et al. [157] reported that the *Capsicum annuum* fruit and seed yields improved when 1% HMWC was put to the ground. According to Krupa & Fornal [158], adding HMWC (970,000 Da) to solid MS media might reduce the salinity stress effect by enhancing the growth of petunia “Prism White” roots and branches. Additionally, Safikhan et al. demonstrated that HMWC application (under concentrations of 0.01% and 0.05%) in milk thistle plants enhanced plants development and growth, elevated proline and soluble carbohydrates, lowered H${}_{2}$O${}_{2}$ content, and improved enzymatic action in leaves under salinity conditions [159]. These findings demonstrated that HMWC may be able to shield plants from the harm caused by salinity stress by increasing the ability of antioxidant enzyme processes.

## 6. Common Presentation

The properties of chitosan can be enhanced by using a variety of methods and broadening the range of its applications. Crosslinking, graft copolymerization, complexation, chemical alterations, and blending are some of the options. An appealing technique that has been widely used to provide chitosan with new desirable qualities is modification through blending. This technique is easy to use, allows combining chitosan with a variety of synthetic and natural polymers, and is useful for everyday use. Regardless of its molecular weight, chitosan can be used as presented below.

### 6.1. In Solution

A quick and easy way to obtain suitable polymeric materials with combined qualities derived from constituent parts for specific purposes is through polymer blending. Blends of natural polymers have recently gained importance because they present a great chance to replace synthetic polymers in many applications and because they are made from renewable resources, are nontoxic, affordable, and their waste is biodegradable. Chitosan and its blends have drawn particular attention among natural polymers because of their adaptability and compatibility for various uses [160,161]. Chitosan properties can be improved by mixing it with both synthetic and naturally occurring macromolecules [162].

Preparation: Chitosan is often blended using one of two main methods: either dissolving it in a solvent and letting it evaporate (solution blending) or mixing it under fusion conditions (melt blending). However, the most widely used technique for creating chitosan blends is solution blending. This is because it is straightforward and suitable for creating different kinds of chitosan mixes (beads, microspheres, films, and fibers). A suitable solvent (often diluted acetic acid) is used to dissolve the chitosan while continuously stirring at room temperature [163]. The next step is to combine with the desired quantity of a different polymer once en dissolution is complete. To enhance its mechanical properties, the chitosan mixture frequently includes a crosslinking agent. After that, the mixture is filtered and cast onto either a petri dish or a glass plate, before it is allowed to cure at room temperature or in an oven. The extra acetic acid must be removed before the mixture is ultimately rinsed with NaOH solution [164].

### 6.2. Hydrogel

The three-dimensional polymeric networks known as hydrogels preserve their structural integrity even on expanding when in contact with water. Because of their great biocompatibility, simple manufacture, and wide range of uses, hydrogels are gaining popularity. Recent years have seen a rise in the development of hydrogels created using diverse biomaterials. Natural biopolymers, particularly polysaccharides like pectin, chitosan, starch, and sodium alginate, are well established as the basic ingredients for the creation of hydrogels to satisfy a range of demands. Chitosan is a food-grade excipient that can be used in encapsulating technology, which is an appealing application [165]. The involvement of two or more compounds in a single complex can serve multiple purposes in plants. Targets that could be effectively exploited in chitosan for applications in plants include metals (e.g., copper, zinc, manganese, and selenium) as well as other biologically active substances like salicylic acid, jasmonic acid, plant secondary metabolites, and essential oils (thymol, neem oil) [166]. This is made possible by chitosan’s mucoadhesive properties, impact on biological surfaces that enhance absorption, simplicity of chemical functionalization, and biocompatibility [165]. Additionally, numerous biological properties of chitosan have been described, including antibacterial properties, which can also constrain the formation of fungal spores, germ tube as well as mycelia. Several studies have suggested that chitosan can be used in food preservation and the packaging industry due to its antimicrobial properties [167]. Chitosan can also be used in wastewater treatment. For example, Jing et al. [168] mixed monomers of tannin and chitosan to prepare a tannin/chitosan/bamboo pulp aerogel adsorbent (TCPA), which played a key role in the efficient and synergistic removal of Cu${}^{2+}$ and Cd${}^{2+}$. This is a green and simple strategy using bamboo pulp, a modified natural poly-saccharide and polyphenol, which has potential in the purification of heavy metal-contaminated wastewater.

Furthermore, as chitosan and anionic molecules interact electrostatically to drive chitosan-based encapsulation mechanisms, the cationic property of chitosan under acidic conditions can be applied in the manufacture of capsules to encapsulate nutrients. Due to its availability, affordability, and abundance of hydroxyl and amino groups, hydrogel beads made of chitosan have gained popularity in recent years for their potential applications in agriculture. Apart from being used as an adsorbent in treating wastewater, chitosan can deliver fertilizers, micronutrients, and herbicides, which have previously shown promising results [165].

Preparation: There are several ways to produce chitosan hydrogels, such as physically joining or chemically crosslinking different geometries and formulas. Typically, chitosan hydrogels are made by dissolving chitosan polymers in dilute acid to create an aqueous solution, which is a classic sol-gel method. After full dissolution, crosslinkers are added to create the hydrogel networks through covalent bonding between polymer chains. To create a network, the crosslinks formed by the crosslinking agent and polymer chains determine the transition solution/gel; these crosslinks do not occur in sufficient numbers in much diluted solutions [169].

A pure chitosan hydrogel can be used in various industries as is or it can be combined with another polymer as an additive. Chitosan creates a cross-linked hydrogel by combining with other naturally occurring polysaccharides such as starch, cellulose, and hemicellulose. Additionally, various synthetic polymers that offer durability, mechanical strength, and elasticity can be combined with chitosan polymers [170].

### 6.3. Chitosan-Based Nanocomposites

NNanomaterials made of chitosan have undergone extensive testing in plants for various qualities, including antimicrobial activity, surface coating and reactive oxygen species (ROS) suppression. Chitosan is used alone or in combination with additional substances such as zinc, silver, and copper to create nanocomposites, which are suitable for all the uses listed before and improve the biological and physical and chemical qualities of chitosan [171]. Additionally, due to their strong antifungal properties, certain nanomaterials are also regarded as viable alternatives to manage phytopathogenic fungi. Due to their remarkable antifungal capabilities, metal nanoparticles, in particular, have been extensively studied, tested and produced substantial results. Metal nanoparticles have been created and utilized to control phytopathogenic fungi in various ways [160]. Metals have a high affinity for chitosan due to chitosan’s polymeric backbone with an abundance of free amine groups [172]. Metals have a high affinity for chitosan due to chitosan’s polymeric backbone with an abundance of free amine groups. Research has mainly concentrated on combining Cu${}^{2+}$ and Zn${}^{2+}$ with chitosan because these two metal ions are crucial for the development and growth of plants. We describe some uses of chitosan-based nanocomposites in plants below.

Antimicrobial activity: Chitosan nanocomposites (Ch-NCs) have antibactericidal activity against either Gram-positive or Gram-negative bacteria. Numerous parameters including MW, DD, concentration and type of acid solvent influence the antimicrobial action of Ch-NCs [173].

Antiviral activity: There are on a few publications on the application of chitosan-based nanomaterials in plant sciences. The antiviral activity of Ch-NCs is unexplored. Fabaceae has a higher Ch-NCs antiviral activity than other botanical groups. Chitosan treatment is highly responsive in beans and peas. Even at high concentrations, low response to Ch-NCs was recorded in the potato, tomato, and tobacco. In the cabbage, endemic diseases including the cauliflower mosaic virus, the radish mosaic virus, and the turnip mosaic virus unsusceptible to chitosan [174].

In seedlings and growth: Chitosan preparations are used strategically by combining them with the right amounts for different plants. Chitosan nanoformulations have a major influence on the behavior, reactivity and toxicity of various plants depending on their kind, preparations in various concentrations and seed priming techniques. As a substantial phytotoxic test, root extension, plant seed germination, and growth vigor index calculation are frequently determined. Cu-chitosan nanoparticles have favorable influences on the dry and wet weight of corn at low concentrations, but at higher concentrations they have negative effects on seedling growth [175,176].

As nanofertilizers: The alternative use of nanobiotechnology emerged as a leader in the creation of unique, ecologically safe nanofertilizer formulas to avoid the indiscriminate use of costly synthetic fertilizers that create pollutants and raise human health and ecological issues. Thus, the most welcome developments in this day and age of highly specialized farming are nano-based smart pesticide delivery systems. At low concentrations, these carriers efficiently, transmit a variety of biologically active nutrients, such as micro- and macronutrients, plant growth regulators, and vitamins [177,178]. The effectiveness of biosynthesized chitosan and Ch-NCs in efficient plant nutrient delivery, absorption, and controlled release mechanisms has been demonstrated in tests using a variety of inorganic mediators including nanoemulsions, nanotubes, metallic nanoparticles, and nanobeads. Ch-NCs have the unique ability to release NPK nanofertilizers to the plants in a controlled manner as they are polycatalytic in nature [179]. In agricultural formulations based on nanotechnology, such as nanopesticides and nanofertilizers, chitosan reacts favorably with negatively charged molecules, ensuring benefits from controlled nanoparticle size. The agricultural industry is also paying close attention to the numerous Ch-NC formulations due to their nanoparticle appeal in illuminating plant growth-promoting activities as well as strengthening and boosting biotic stress tolerance qualities. The creation of a core material for encapsulation with adequate physicochemical surface qualities, size, and biocompatibility is a subject of extensive research [171].

## 7. A Perspective of the Authors

As our review work indicates, the potential of chitosan in agriculture is indisputable; however, we can ask the following question: How does chitosan fit in with emerging trends and technologies? The answer may lie in the word nanotechnology. Nanotechnology has allowed development of methodologies that facilitate development of more efficient products. For example, with the use of nanotechnology we can obtain chitosan nanoparticles which have high bioavailability and able to encapsulate micro and nano elements, nutrients, and nanoparticles. Thus, nano fertilizers can be formulated, which have high bioavailability, low environmental impact, high soil fertility, productivity, and high quality of agricultural products. This review is only a perspective of the authors but as we continue to study the subject, new questions will be generated and opportunities for continuous evolution in agriculture will arise.

## 8. Conclusions

The mechanism of action of chitosan depends mainly on the molecular weight and DD. Chitosan commonly dissolves in acidic media resulting in a high concentration of protons in the medium, which then protonatethe amino group (NH${}_{2}$). Therefore, chitosan can easily react with cell membranes of negatively charged bacteria. The molecular weight of chitosan plays an important role in this mechanism, as the size of the polymeric chains depends on the molecular weight, the one with the lowest molecular weight having the greatest availability of amino groups. For example, for high molecular weight chitosan, it could can form a superficial polymeric membrane in covering the bacterial cell, thereby blocking nutrients and causing inhibiting bacterial growth. This same mechanism is more pronounced in medium molecular weight chitosan as it has a greater availability of charges giving a greater stability in the cover formed causing cell lysis. Both HMWC and MMWC have a preferential effect on Gram-positive bacteria due to their cell wall components but have no effect on Gram-negative bacteria.for the mechanism of action of low molecular weight chitosan differs somewhat. In addition to to forming a superficial film (in this case the film would be thinner), it can also penetrate the cell wall of the bacteria and distrupt its genetic material thereby inhibiting bacterial growth. In summary, chitosan in its low molecular weight form inhibits Gram-negative bacterial growth and in its medium and high molecular weight can inhibit Gram-positive bacteria. Figure 2 summarizes the mechanisms of action of chitosan. There is a correlation between the mechanism of action of chitosan and the one described in Section 5.4.1. However, the mechanism can also vary depending on the condition. In addition to the aforementioned mechanism of action, another mechanism is that chitosan can enter the cell and interact with the phosphate group, alter DNA, and cause oxidative stress. Based on the studies reviewed here on the effect of chitosan as a plant growth promoter, the following main conclusions can be made: that chitosan activates hydrolytic enzymes to degrade and mobilize reserve food materials, and that it promotes root cell division by activating plant hormones such as auxin and cytokinin. This has a direct effect on nutrient adsorption [91]. The major findings also indicate that low density chitosan is most suitable for use as growth promoter. This review has also indicated that the use of chitosan must be in low concentrations; otherwise it will have an adverse effect and inhibit root growth. This is demonstrated by the fact that chitosan in high concentrations causes the accumulation of auxin, which reduces the length of the primary root and altering the sprouting of secondary roots.

## Figures and Tables

**Table 1:** Main origins of chitosan or chitin in both terrestrial and marine organisms [20].

Marine
**Sellfish**
Crab
*Chionoecetes opilio* [21]
*Podophthalmus vigil* [22]
*Paralithodes camtschaticus* [23]
*Carcinus mediterraneus* [24]
**Water lobster**
Crawfish [25]
**Shrimp**
*Aristens antennatus* [26]
**Krill**
*Daphnia longispina* [27]
*Anax imperator* [28]
*Hyrophilus piceus* [28]
*Notonecta glauca* [28]
*Agabus bipustulatus* [28]
*Asellus aquaticus* [28]
**Molluscs**
*Squid pens*
*Loligo* sp. [29]
*Todarodes pacificus* [30]
**Terrestrial**
**Arthropods**
Spyders
*Geolycosa vultuosa* [31]
*Nephila edulis* [31]
**Scorpions**
*Mesobuthus gibbosus* [32]
**Beetles**
*Bombyx mori* [33]
*Holotrichia parallela* [34]
*Leptinotarsa decemlineata* [35]
Cockroaches [36]
**Brachiopods**
*Lingula seta* [37]
**Microorganisms**
**Fungus (cell walls)**
Ascomydes
*Mucor rouxii* [38]
*Blastomycota*
*Blastocladiaceae* [39]
*Chytridiomycota*
*Chytridiaceae*
**Protista**
Brown algae [40]
**Plantae**
Green algae [40]

**Table 2:** Chitosan properties by Molecular-Weight.

Chitosan			
**Properties**	**Low** **Molecular Weight** **(>150 kDa)**	**Medium** **Molecular Weight** **(150–700 kDa)**	**High** **Molecular Weight** **(>700 kDa)**
**Physicochemical**	High nitrogen content		
	Weak base with deprotonated amino groups as nucleophiles (pKa = 6.3)	Medium nitrogen content	Low nitrogen content
	Great reactive groups for crosslinking and chemical activation	Limited solubility	Limited solubility
	Has chelating and complexing properties	Pseudoplastic behavior	Pseudoplastic behavior
	Ionic conductivity as polyelectrolytes (pH < 7)	Good reactive groups for crosslinking and chemical activation	Low reactive groups for crosslinking and chemical activation
	Great solubility	High tensile strength	Low tensile strength
	Low viscosity	Medium viscosity	High viscosity
	High permeability	Medium permeability	Low permeability
	Low melting point		
	High tensile strength		
**Biological**	Biocompatible		
	Nontoxic		
	Biodegradable		
	Antimicrobial (fungi, bacteria, viruses)	Biocompatible	Biocompatible
	Mucoadhesive	Nontoxic	Nontoxic
	Antioxidant	Antimicrobial (fungi, bacteria, viruses)	Biodegradable
	Antitumor	Higher antioxidant	Antimicrobial (fungi, bacteria, viruses)
	Hypolipidemic		Lower antioxidant
	Blood anticoagulants		
	Hypocholesteromic activities		

**Table 3:** Chitosan’s inhibitory effects on different fungal pathogens [90].

Pathogen	Chitosan Molecular Weight	Chitosan Content in Acetic Acid	Research Strategy	Bacteriostatic Result	Reference
*Botrytis cinerea*	97% of deacetylation degree and 7.6 × 10${}^{3}$	0.5 and 1 mg/mL	Potato dextrose agar (PDA)	-	[121]
*Escherichia coli*	82.33% of deacetylation degree and 1.21 × 10${}^{6}$	1 mg/mL	Growth medium	85%	[86]
*Fusarium graminearum*	2.8 kDa	1 mg/mL	Agarose culture	-	[122]
*Magnaporthe grisea*	13.4 to 18.8% acetylation degree and average molecular weight of 10 kDa	0.5 mg/mL	Agar (HiMedia)	57%	[123]
*Alternaria solani*	2 to 61% of deacetylation degree and average kDa of 42.5 to 135	25 mg/mL	PDA and Muller-Hinton agar	24%	[124]
*Xanthomonas oryzae*	607 kDa	2 mg/mL	PDA	76.47%	[125]

## Data Availability

The data presented in this study are available on request from the corresponding author.
